# Dissecting Neuronal Activation on a Brain-Wide Scale With Immediate Early Genes

**DOI:** 10.3389/fnins.2020.569517

**Published:** 2020-10-23

**Authors:** Alessandra Franceschini, Irene Costantini, Francesco S. Pavone, Ludovico Silvestri

**Affiliations:** ^1^European Laboratory for Non-linear Spectroscopy (LENS), Sesto Fiorentino, Italy; ^2^National Institute of Optics, National Research Council (INO-CNR), Sesto Fiorentino, Italy; ^3^Department of Physics and Astronomy, University of Florence, Florence, Italy

**Keywords:** immediate early genes, tissue clearing, light-sheet microscopy, high-throughput microscopy, image analysis, whole-brain mapping

## Abstract

Visualizing neuronal activation on a brain-wide scale yet with cellular resolution is a fundamental technical challenge for neuroscience. This would enable analyzing how different neuronal circuits are disrupted in pathology and how they could be rescued by pharmacological treatments. Although this goal would have appeared visionary a decade ago, recent technological advances make it eventually feasible. Here, we review the latest developments in the fields of genetics, sample preparation, imaging, and image analysis that could be combined to afford whole-brain cell-resolution activation mapping. We show how the different biochemical and optical methods have been coupled to study neuronal circuits at different spatial and temporal scales, and with cell-type specificity. The inventory of techniques presented here could be useful to find the tools best suited for a specific experiment. We envision that in the next years, mapping of neuronal activation could become routine in many laboratories, allowing dissecting the neuronal counterpart of behavior.

## Introduction

The most direct readout of brain activity is behavior. Although there is a consensus about the fact that our actions are a result of the coordinated activity of our neurons, the causal links between these two phenomena are still largely unknown. Understanding how neuronal networks in the brain drive specific behaviors, and how these networks change with experience is thus a fundamental challenge of neuroscience. In addition, a deeper insight into the connection between brain activity and behavior would also shed light on the mechanisms that disrupt this link in pathology, laying the basis for better treatment of mental diseases.

From a methodological point of view, understanding this connection requires techniques for whole-brain mapping. Indeed, neuronal activation patterns should be studied on the same scale of the structural organization of neuronal networks, i.e., brain-wide. One would ideally need some methods to record *electrical activity simultaneously* from *all* the neurons in the brain of a *freely behaving subject*, with *single-cell resolution*. It is immediately apparent that no technique with such capabilities exists, and even if we relax, some of the requirements in italics, whole-brain mapping still sounds like a formidable task for state-of-the-art technologies. Traditional functional imaging methods used to reveal large-scale neuronal activity, like functional magnetic resonance imaging (fMRI) or electroencephalography (EEG), lack the proper spatial resolution to record single-cell activity (Logothetis, 2008; Michel and Brunet, 2019). On the other hand, single-cell electrophysiology cannot scale to more than a handful of neurons (Perin and Markram, 2013).

Over the last decade, *in vivo* optical methods have demonstrated the ability to work across different scales (Carrillo-Reid et al., 2017; Yang and Yuste, 2017; Sancataldo et al., 2019), allowing registration of membrane potential or of intracellular calcium in hundreds or even thousands of neurons simultaneously (Ahrens et al., 2013; Prevedel et al., 2014; Nöbauer et al., 2017). However, these techniques still suffer several limitations:

1. There is a practical trade-off between spatial resolution and field of view (FOV). For instance, calcium imaging of the entire mouse cortex can be achieved only at coarse resolution (Vanni et al., 2017), while single-cell recordings are limited to a smaller spatial area (Prevedel et al., 2014).
2. Light scattering by nervous tissue limits imaging penetration to less than an mm (Helmchen and Denk, 2005). Thus, in rodents, *in vivo* optical imaging is limited to the cortex [unless endoscopic approaches are used (Dombeck et al., 2010)]. Whole-brain imaging has been achieved hitherto only in small organisms like the nematode *Caenorhabditis elegans* or the larva of *Danio Rerio* (zebrafish) (Ahrens et al., 2013; Prevedel et al., 2014).
3. Optical microscopes are usually quite sophisticated and heavy, and thus hardly compatible with freely behaving animals. Simpler systems—with poor resolution—can be used on mice moving freely in their cage (Aharoni et al., 2019). However, when single-cell resolution is sought, the only acceptable compromise is to place a head-fixed animal in a virtual reality environment (Dombeck et al., 2010).

**FIGURE 1:**
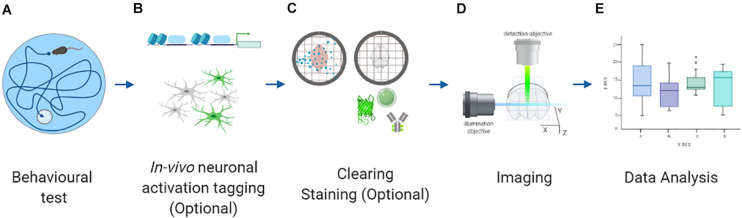
Scheme representing the different steps to perform whole-brain neuronal activation mapping. **(A)** Behavioral testing. **(B)** *In vivo* neuronal activation tagging with endogenous fluorescence proteins. **(C)** Sample preparation protocol: clearing and staining (optional, when the tagging is not performed). **(D)** Imaging with advanced fluorescence microscopy. **(E)** Data analysis with different algorithms. Created with BioRender.com.

To circumvent these constraints, a radical solution is to tag activated neurons *in vivo* and image them subsequently *ex vivo* (Figures 1A,B). Fixed murine brains can be cleared and labeled using many different protocols (Figure 1C). Afterward, a comprehensive yet high-resolution reconstruction of these samples can be obtained using the latest developments in *ex vivo* microscopy (Figure 1D). Finally, quantitative data can be extracted from raw images using state-of-the-art algorithms (Figure 1E). These inherent advantages have been exploited to quantify brain-wide neuronal activation by targeting neurons expressing immediate early genes (IEGs). These genes are expressed by neurons under sustained activation (Bartel et al., 1989) and are considered a reliable proxy for activity. Indeed, IEG quantification is a classical method to study neuronal activation in selected brain areas (Morgan et al., 1987; Sagar et al., 1988).

*Ex vivo* whole-brain mapping of IEGs eventually allows to *map activated neurons* across *the entire brain* of a *freely behaving subject*, with *single-cell resolution*. In the end, this is not too far from the original challenge we issued a few lines before.

In the following, we review the latest technological developments concurring to map and manipulate activated neurons across the entire mouse brain. This ambitious goal is today possible by combining advancements from multiple fields, including genetics, tissue preparation, optical imaging, and image analysis. The purpose of our review is to guide the reader into this multidisciplinary research, offering the tools to choose the techniques that are best suited for a specific application. For detailed recapitulation of the single topics, we refer to excellent reviews that have been published in the last years (Mayford and Reijmers, 2016; Peng et al., 2016; Sbalzarini, 2016; Tainaka et al., 2016; DeNardo and Luo, 2017; Power and Huisken, 2017; He et al., 2019; Ueda et al., 2020).

## Tagging Neuron Activation Following Sensory and Behavioral Stimuli

The first step to map activated neurons across the whole brain is to tag them with a label allowing visualization through an optical microscope. Different methods have been proposed during the years, ranging from classical immunohistochemistry to sophisticated transgenic or viral approaches. All these different techniques share a common principle: the use of immediate early genes (IEGs). In this section, we first recall the basic features of IEGs and then describe in detail the various methods that could be used to tag neurons according to the expression of one of these genes.

### IEG-Based Approaches as Useful Tools to Access Activated Neurons

Nowadays, the discovery of IEGs has enabled us to reconstruct functional maps with single-cell resolution. IEGs, such as *c-fos*, *Arc*, and *Egr1* (also known as *ZiF268)*, are a class of genes that is activated transiently and rapidly in response to a wide variety of cellular stimuli (Hunt et al., 1988; Sagar et al., 1988; Kaczmarek and Nikolajew, 1990; Hughes et al., 1992; Pinaud, 2004; Terleph and Tremere, 2006; Bahrami and Drabløs, 2016). At the brain level, the expression of all IEGs is induced by neuronal activity through depolarization (Greenberg et al., 1986; Morgan et al., 1987; Bartel et al., 1989; Sheng and Greenberg, 1990; Smeyne et al., 1992). In this way, the rise of intracellular Ca^2+^ levels activates second messenger pathways that, in turn, stimulate transcriptional factors. Within few minutes, these factors trigger the expression of the relative genes. For that reason, for a long time, they have been used as an indirect marker to measure neuronal activity. In addition to their rapid induction, IEG proteins have a relatively short life due to their fast transcription that is interrupted with the end of external stimulation. Thus, in a few hours, the expression levels of these proteins return to their baseline (Sheng and Greenberg, 1990).

These genes respond to a wide variety of intrinsic and extrinsic stimuli, including growth factors, high intracellular levels of Ca^2+,^ and cAMP, strong depolarization, receptors activating, and neurotransmitters, indicating a very general response mechanism (Bartel et al., 1989; Sheng and Greenberg, 1990; Ghosh et al., 1994; Fowler et al., 2011). Regulation of gene transcription varies according to the type of gene and brain region (Sheng and Greenberg, 1990; Mayford and Reijmers, 2016). For example, growth factors and membrane depolarization activate distinct programs of early response gene expression (Bartel et al., 1989). Cole et al. (1989) found that a rapid increase in IEGs due to neuronal stimulation has a critical role in long-term change in synaptic efficiency. They proved that *in vivo* stimulation of DG hippocampal cells with electrodes was sufficient to produce long-term potentiation (LTP), causing the induction only of Egr1, while c-fos needed more prolonged stimulation (Cole et al., 1989; Sheng and Greenberg, 1990). In both cases, the IEG activation was correlated to the elevated presence of NMDA receptors (Morgan et al., 1987; Sheng and Greenberg, 1990).

Starting from these characteristic features of IEGs, a series of IEG-based tools have been developed either in the form of transgenic mice or viral vectors (Reijmers et al., 2007; Guenthner et al., 2013; Kawashima et al., 2013; Sakurai et al., 2016; Sørensen et al., 2016; DeNardo et al., 2019; Hasan et al., 2019). These genetic approaches have been developed to translate neuronal activity into gene expression by making possible the visualization of behavioral-relevant cells and also the access to the same neurons in living animals or *ex vivo* tissue slices. Most of these strategies pave the way to cell manipulation with chemogenetic or optogenetic tools, giving to researchers the possibility to activate, inactivate, and record neuronal activity using electrophysiology or genetically encoded calcium indicators (Barth et al., 2004; Wang et al., 2006).

Before describing in detail the mechanisms, advantages, and disadvantages of each genetic approach, it is worth adding a few general considerations. IEG transcripts or proteins are present in neurons for a limited time after the stimulus that elicited the activity of a specific brain circuit. Therefore, any protocol aimed at mapping the presence of IEGs must be performed within a limited time window after the behavioral test. If proteins or transcripts have to be mapped directly, e.g., with immunohistochemistry (IHC), this means that the animal must be sacrificed after the stimulus, preventing further behavioral tests. The limitation of IHC is related to IEG kinetics because its peak expression and the return to baseline are very rapid (Morgan et al., 1987). Genetic approaches, driving recombination in the presence of IEGs, have been developed to overcome this limitation allowing access to activated cells for a period much longer than the IEG timescale, even 1 month later (DeNardo et al., 2019).

The time window of IEG expression also defines the maximum temporal resolution of the method, i.e., the capability to distinguish neurons activated by the stimulus of interest from those activated by subsequent or previous stimuli. When using transgenic or viral strategies in combination with some drug, the pharmacokinetics of the drug itself must also be taken into account, as it will often extend the time window beyond the natural half-life of IEG proteins (Sheng and Greenberg, 1990; Reijmers et al., 2007; Guenthner et al., 2013). For instance, Cre-based recombination systems isolates activated cells with a time resolution similar to that of endogenous IEG expression (few hours) (DeNardo et al., 2019), while in some approaches based on Tet-tag, the half-life of the drug determines a time window of few days (Reijmers et al., 2007). This loss of temporal resolution leads to the labeling of more neurons than those activated in the behavior of interest. These “false positives,” usually referred to as “background,” effectively reduce the capability of the method to find statistically significant variations in the number of activated neurons between different areas or subjects.

On the contrary, “false negatives” can appear when the method fails either to tag-activated cells or to maintain labeling after the recombination event (as in the case of genetic methods). In both cases, a fraction of IEG-positive neurons is missing, again reducing statistical power. The main reasons for the insufficient labeling of activated cells are limited efficiency of the recombination mechanisms in selected cell types or also the limited penetration of exogenous dyes (e.g., in IHC).

**FIGURE 2:**
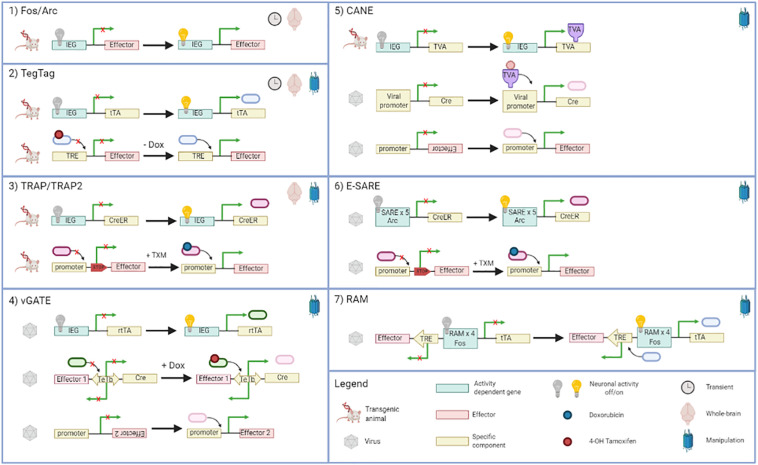
Diagram of immediate early gene (IEG)-based approaches for neuron tagging. For each method, information about the characteristic of the final effect is described. In particular: the duration time (transient or not), the location (whole-brain or not), and if the method can be coupled with optogenetic and chemogenetic tools (manipulation). TRE, tetracycline responsive element; Dox, doxycycline; tTA, artificial transcription factor; TXM, 4-hydroxytamoxifen; CreER, tamoxifen-inducible recombinase; rtTA, reverse tetracycline transactivator; TeTb, bidirectional Tet promoter. Created with BioRender.com.

Here below, we describe in detail the existing methods able to tag activated neurons, providing useful information for researchers. Figure 2 summarizes them with schematic illustrations, while Table 1 recapitulates the main features of each method.

**TABLE 1:** Summary of the main features of each immediate early gene (IEG) tagging method.

	IHC	Fos-GFP Arc-dVenus	TetTag	TRAP	TRAP2	CANE	vGATE	E-SARE	RAM
Whole-brain cell counts	X	X	X	X	X				
Projection mapping of activated cells				X	X	X	X	X	X
Tran-synaptic tagging				X	X	X			
Optogenetic or chemogenetic manipulation of activated cells			X	X	X	X	X	X	X
Subsequent behavioral test				X	X	X	X	X	X
Cell-type specificity							X		

*X, Means that this IEG tools has that feature.*

#### Immunohistochemistry

The immunohistochemical staining has been considered the “gold standard” for the reconstruction of whole-brain activity maps at the cellular scale. Leveraging the fact that IEG synthesis is induced by neuronal activity, the first way to visualize the activity at the brain level was obtained by the immunostaining of *c-fos* (Morgan et al., 1987; Sagar et al., 1988). Despite the large number of genetic approaches developed in the last years, IHC still remains the reference method to map IEG-expressing cells. It has been coupled with many clearing techniques, such as iDISCO, CLARITY, CUBIC, for the 3D reconstruction of brain-wide activated circuits (Susaki et al., 2014; Tomer et al., 2014; Renier et al., 2016). However, there are some limitations concerning this method. After the stimulus, when IEGs arrive at the transcription peak, animals have to be sacrificed, so it is not possible to carry out other studies on the same animal.

Moreover, it is often interesting to understand whether a neuronal ensemble, activated with a stimulus, could be reactivated with a distinct one, and the traditional immunohistochemistry does not allow this type of investigation. In order to solve this latter problem, *cellular compartment analysis of temporal activity by fluorescent in situ hybridization* (catFISH) has been developed. This technique exploits different compartmentalization of mRNA into the nucleus and the cytoplasmic region (Guzowski and Worley, 2001). However, starting from the previous method, a new double-labeling technique is applied in order to study two sequential stimuli separated by an appropriate interval. Thus, the *tyramide-amplified ICH-FISH* (TAI-FISH) exploits different compartmentalization of IEG protein and the relative mRNA, in this way the first stimulus is visualized by IEG cytoplasmic protein while the second with the mRNA in the nucleus (Xiu et al., 2014).

#### Fos-GFP and Arc-dVenus

As mentioned above, IEGs offer genetic access to neurons activated in response to specific stimuli; therefore, strains of transgenic or knock-in mice have been developed to directly link IEG transcription to some type of reporter. Genes coding for fluorescent proteins, LacZ markers or luciferase, are inserted under the control of the IEG-promoters (Smeyne et al., 1992; Barth et al., 2004; Wang et al., 2006; Xie et al., 2014; Kim Y. et al., 2015; Vousden et al., 2015). This approach has been first proven in transgenic mice in which *Fos* promoter drove transcription of the β-galactosidase; neurons activated in response to light pulse stimulation during the dark cycle were then identified as Fos-LacZ positive through a blue pigment (Smeyne et al., 1992). More recently, several transgenic models have used fluorescent proteins as reporters in an activity-dependent manner (Barth et al., 2004; Wang et al., 2006; Xie et al., 2014; Kim Y. et al., 2015; Vousden et al., 2015). For example, *Fos-GFP* mice have been used to map the neurons stimulated during social or parenting behaviors (Kim Y. et al., 2015), while different activation patterns involved in recalling contextual and tone fear memories at whole-brain level were explored with the *Arc-dVenus* strain (Vousden et al., 2015). The half-life of the fluorescent proteins is similar to endogenous IEGs ones, leading to fluorescent peaks in a short time, about 1–2 h, and lasts approximately 6–8 h. This temporary access to neurons prevents manipulation of the same activated cells later in time. Still, GFP-expressing cells may be targeted *in vivo* for electrophysiological recording right after the behavioral experiment (Barth et al., 2004). *Fos-GFP* and *Arc-dVenus* have been included inside a pipeline that demonstrated, for the first time, the possibility to detect neural activity on a brain-wide scale, using automated whole brain imaging (Kim Y. et al., 2015; Vousden et al., 2015).

#### TetTag

More complex strategies limit the expression of an IEG-linked effector to some kind of pharmacological treatment. One of the first approaches demonstrated in this sense is *TetTag*, which uses *Fos* promoter to drive the expression of a doxycycline-repressible tetracycline transactivator (tTA) (Reijmers et al., 2007). This strategy has been developed to understand whether neurons activated during fear learning are reactivated during fear memory recall (Reijmers et al., 2007). This inducible expression system, called *Tet-Off*, exploits the “switch-off” mode in the presence of the doxycycline antibiotic (DOX). The *TetTag* strategy needs two transgenes: the first requires the tTA, an artificial transcription factor while the second, a tetracycline-responsive element (TRE) that is a synthetic promoter. TRE needs to bind to tTA for the expression of any genes. During the resting state, tTA is usually bound to DOX and consequently unable to link to the TRE sequence. Therefore, in the presence of a stimulus and of a diet lacking in DOX at the same time, the *Fos* promoter stimulates the synthesis of tTA, which now is able to bind TRE for the effector expression, achieving neuronal labeling. Due to the slow metabolism of DOX, the time window becomes very wide and leads to high levels of neuronal background (Shockett and Schatz, 1996; Reijmers et al., 2007). Moreover, this method does not allow to permanently access to activate neurons because TRE-conditional effectors last only few days (Reijmers et al., 2007).

Finally, for the manipulation of behavioral-responsive ensembles in many paradigms, this technique can be coupled with optogenetic and chemogenetic tools, using some rhodopsins and excitatory human M3 muscarinic (hM3Dq) receptors directly as effectors (Koya et al., 2009; Garner et al., 2012; Liu et al., 2012; Ramirez et al., 2013, 2015; Cowansage et al., 2014; Redondo et al., 2014).

#### TRAP

*Targeted recombination in active populations* (TRAP) is another drug-dependent approach, such as *TetTag.* However, compared to the latter, TRAP shows a better temporal resolution and permanent genetic access to neurons, making activated cells permanently fluorescent (Guenthner et al., 2013). Like the previous one, also this strategy needs two transgenes: the first expressing a tamoxifen-inducible recombinase CreER^*T*2^ in an activity-dependent way, i.e., under the control of *Arc* and *Fos* promoter (ArcTRAP and FosTRAP) (Feil et al., 1997). The second exploits the Cre-Lox recombination under a ubiquitous promoter for the reporter expression.

CreER^*T*2^ is expressed in active cells but is not effective unless in the presence of tamoxifen (TMX). When present inside cells, TMX binds the ER site allowing CreER^*T*2^ to move from the cytoplasm to the nucleus, driving the expression of a reporter (*i.e., fluorescent protein*) by the removal of loxP-stop-loxP sequence (Guenthner et al., 2013). The time window for “*cell TRAPing*” is provided by the lifetime of TMX and, therefore, by its metabolism and excretion. Due to the long lifetime of TMX, Guenthner et al. (2013) have decided to use its metabolic form, 4-hydroxytamoxifen (4-TMX), limiting the time window to a period <12 h. As a result, only neurons that are activated around drug administration can be TRAPed.

This genetic tool has facilitated previously impossible experiments, enabling the manipulation of neural ensembles activated during a specific task later in time, even days after (Ye et al., 2016; Ishii et al., 2017; Kim and Cho, 2017; Girasole et al., 2018). Moreover, this method has allowed whole-brain reconstructions and it is often coupled with clearing approaches. Besides the many progresses made in this field, TRAP has some limitations: the first is caused by the *Arc* and *Fos* haploinsufficiency that provokes the disruption of their endogenous expression; the second is about stochastic labeling mainly due to random reporter expression.

#### TRAP2

Recently, *TRAP* has been improved with a new version, called *TRAP2* (DeNardo et al., 2019). The mechanism is the same as *TRAP* with few but essential developments. *TRAP2* is further optimized with an improved Cre (iCre) to enhance the effector expression (Shimshek et al., 2002). It preserves the Fos endogenous expression, allowing keeping transgenic animals in homozygosis. Therefore, this capability of maintaining Fos endogenous expression also improves the penetration in many brain regions. Using the *TRAP2* tool, *Luo’s group* has studied fear memory retrieval in the prelimbic cortex.

#### CANE

Sakurai et al. (2016) have developed a “lock-and-key strategy” for *capturing activated neuronal ensembles with engineered mice and viruses (CANE)*. In the *CANE* system, a destabilized targeting avian leukosis (dsTVA) receptor, not present in mice, is knocked-in to endogenous Fos locus. In the presence of external stimuli, Fos and dsTVA are co-translated in the same neuron so that all Fos^+^ cells have on their membrane the avian receptors. Before any external stimulation, rabi/lentiviruses coated with a surface glycoprotein (EnvA), a typical ligand of TVA, are delivered into the brain and infect Fos^+^ neurons in the injection area. After transfection, the virus genome is inserted into the neuron and allows the effector expression (Sakurai et al., 2016).

CANE leads to a more precise temporal and spatial resolution compared to previous techniques. Its short time window depends a) on the brief half-life of dsTVA, which mimics the kinetics of the endogenous *Fos*, and b) on tightened limitations of viral vectors transduction. For those reasons, it enables low background and it has been used to study “mild behavior” (*i.e., brief behavioral encounters or brief behavioral events*). Moreover, this double-control mechanism appears to permanently tag the majority of activated cells, leading to high efficiency. The use of lenti and rabiviruses allows the labeling of two or more ensembles in the same brain region and also *trans-*synaptic tracing of activated cells (Sakurai et al., 2016; Rodriguez et al., 2017; Jiang-Xie et al., 2019; Tschida et al., 2019). CANE is an excellent technology, able to reconstruct efferent and afferent connections, but it cannot be used for whole-brain labeling because viral infection is limited to the injection site. Moreover, virus delivery requires stereotaxic surgery and anesthesia, which could alter behavior and neuronal response.

#### vGATE

The most recent genetic approach, which uses a mixture of three viruses, is called *virus-delivered genetic activity-induced tagging of cell ensembles* (vGATE). This multilevel strategy has been used to investigate fear memory engrams and especially to manipulate hypothalamic oxytocin neurons with the aim of understanding the role of this subpopulation in fear response (Hasan et al., 2019). The authors used a system composed of three adeno-associated viruses (AAV). The first one drives the expression of a reverse tetracycline transactivator (rtTA) under the *Fos* promoter. In order to have a permanent tagging of Fos^+^ neurons, a Tet operator sequence has been integrated upstream of *Fos* promoter, able to sustain an extensive induction of rtTA in the presence of DOX through an autoregulatory expression loop. The second virus contains a bidirectional Tet promoter that, in the presence of the DOX, stimulates the expression of a fluorescent protein and simultaneously activates the Cre recombinase. The last virus uses a cell-type-specific promoter that, under a Cre recombinase, expresses Channelrhodopsin-2 (ChR2) to optically manipulate neuronal activity (Hasan et al., 2019).

Contrary to *TetTag*, this IEG-based method exploits a Tet-On system, i.e., DOX “switch-on” mode. By the intraperitoneal DOX injection, the transcriptional activator is expressed, providing a better-controlled time window, which depends only on the drug metabolism and viral transduction, avoiding the integration of the DOX in the feed.

The great advantage of the vGATE is the lack of use of transgenic mice; in this case, there is the possibility to switch to other species. For instance, Hasan et al. (2019) have applied this tool to rats for their study. Moreover, the last virus contains a cell-type-specific promoter, and consequently, a specific neuron subpopulation may be manipulated and visualized. The use of viruses has its own limitations, yet it cannot be used for systemic tagging due to the local nature of viral injection; furthermore, as always in a complex stereotaxic surgery, the potential effects of any brain injury on animal behavior could not be overlooked.

#### E-SARE

Recently, many researchers focused on endogenous promoters, modifying their genomic sequences to have better control of the transcription of IEGs induced by neuronal activity. Therefore, viral strategies using engineered promoters have expanded the horizon of IEG-based methods to improve the specificity and efficiency of activated neurons, increasing reporter expression level more than 20-fold. Kawashima et al. have introduced the *synaptic activity-responsive elements* (SAREs), which regulate *Arc* expression throughout the cooperation of three activity-dependent transcription factors (CREB, MEF2, and SRF) to induce a strong transcription (Kawashima et al., 2009). Exploiting their study based on the SARE enhancer element of the Arc promoter, the same authors have constructed a new synthetic promoter called “E-SARE” that is composed of five tandem repeats of SARE sequence fused into an *Arc* minimal promoter (Kawashima et al., 2013). This genetic tool has been used to tag neurons activated by different visual orientation stimuli. In order to obtain a reliable circuitry map and to manipulate permanently tagged neuronal ensembles, E-SARE has been coupled to an inducible Cre sequence. The combination of the E-SARE upstream of the CreER^*T*2^ allows to have a tightly controlled time resolution (Kawashima et al., 2013). This system can be packed into AAV vectors or lentivirus without the use of transgenic animals; consequently, the great advantage is the potential “switching” to larger mammalian species.

#### RAM

*Robust activity marking* (RAM) system is another method, such as E-SARE, that exploits a synthetic promoter to investigate activity-dependent cells. This strategy has been used to label and manipulate neuronal ensembles in the hippocampus of animals subjected to contextual fear conditions (CFC) and in the amygdala following tone-fear conditioning (TCF) (Sørensen et al., 2016). The RAM promoter (P_*RAM*_) is composed of four repeats of an enhancer module, which is composed by the AP-1 site and the neuronal-specific activity-dependent gene Npas4-binding motif, upstream of *Fos* minimal promoter (Sørensen et al., 2016). Related to existing IEG-genetic tools that do not take advantage of synthetic promoters, RAM has been developed to reduce background levels, meaning that it only responds to neuronal activity with a robust signal and to precisely control the time of its activation. P_*RAM*_ has been combined with an improved version of a Tet-Off system with a destabilized version of tTA (d2tTA) for many reasons: best activation time and tight time-window that leads to lower basal expression. Moreover, the P_*RAM*_ small dimension allows to pack the entire DNA sequence into a single virus, bypassing transgenic animals and using it in other species like rats or flies, thanks to high conservation of the sequence used (Sørensen et al., 2016). Finally, to show the versatility of this method, a Cre-dependent RAM has been developed to study cell type-specific ensembles activated by a specific stimulus (Sørensen et al., 2016).

### Different Approaches, Not Based on IEG, to Tag Activated Neurons

IEG-based approaches have been generally used to tag neurons activated during behavioral experiences. Still, these methods cannot be used to study “mild” behaviors or behaviors that produce neuronal activity less sustained in time. The reason lies in the fact that these IEGs share some limitations, which are mainly caused by the long time-window, ranging from hours to days, and high-level background. For that reason, photoactivatable approaches have been developed to overcome the problems related to IEG’s nature, using calcium as indicator for neuronal activity and light instead of drugs, as tool for a more rapid temporal resolution and better control of non-specific reporter expression, significantly lowering the background. FLARE (Wang et al., 2017) and Cal-Light (Lee et al., 2017) are the first Ca^2+^-and-light-gated tools that exploit a transcriptional readout, while CaMPARI (Fosque et al., 2015) is another Ca^2+^-and-light-gated tool that uses fluorescent protein photoconversion.

## Tissue-Clearing Protocols as Fitting Tools to Image Three-Dimensional (3D) Brain Volume

Once neurons are activated and labeled in response to behavioral stimuli, brain samples can be analyzed with optical microscopy. However, given the opaque nature of biological specimens, the full volume of the brain cannot be directly reconstructed in 3D. From standard histology to more recent techniques, methods based on serial sectioning are able to reconstruct the 3D volume (for more details, see section “Quantifying Neuronal Activation Across the Entire Murine Brain”). These methods are based on mechanical operations that lead to sample disruption. Indeed, the brain cutting could cause compression, stretching, or accidental incision, which often make the volumetric reconstruction hard. Although these approaches are widely used, the most straightforward way to preserve the 3D structure is to make specimens transparent through tissue clearing methods. The transparency provides direct optical access to bulky specimens, allowing to overcome sample sectioning. In general, keeping brains intact rather than exploring smaller parts is important to achieve a better comprehension of neuronal mechanisms. It is evident that virus injections, generally used to tag neuronal projections of neuronal subgroups activated by a particular stimulus, or used to select specific subpopulations, could be scarcely evaluated by two-dimensional (2D) sections. Neuronal projections are extended in every possible direction; therefore, brain cutting can result in loss of information about connections between regions or about the virus pathway.

**FIGURE 3:**
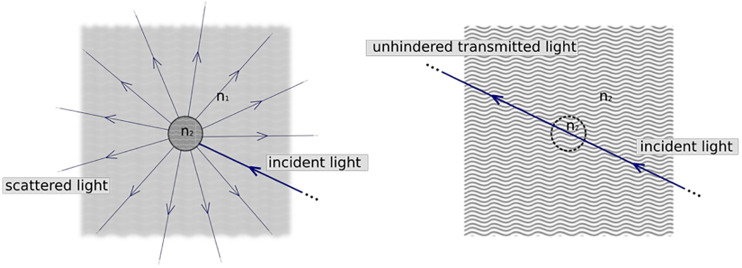
Physical principle underlying tissue clearing. In normal tissue **(left)** cellular components have a refractive index n_2_ significantly larger than that of the surrounding medium (water, n_1_). This inhomogeneity results in scattering of light and thus opaqueness of the sample. Clearing methods try to match refractive indices **(right)**, so that tissue appears as a homogeneous optical medium where light can travel unhindered. Created with BioRender.com.

As mentioned above, a relevant problem to rapidly image large volumes of tissue is associated with the milky aspect of the brain due to its heterogeneous composition. This heterogeneity leads to light scattering, with light rays diffused in random directions by the microscopic components of the sample. This diffusion of light hinders brain imaging. Indeed, light is scattered as a result of the mismatch between the refractive indices (RIs) of different tissue components (see Figure 3). Possible solutions to overcome this problem consist of limiting the scattering effects by reducing optical inhomogeneities within the sample (Tuchin, 1997; Richardson and Lichtman, 2015). Thus, the clearing protocols work by minimizing the mismatch between macromolecules and the surrounding medium. Generally, the “dry” part of biological tissue (proteins and lipids) has a high RI (ranging from 1.4 to 1.6), while the surrounding medium is mainly composed of water, which has an RI of 1.33 (Jacques, 2013). The first approach to achieve the transparency of the brain is related to the removal of lipids that are the primary source of scattering in the fixed sample. By eliminating lipids from the sample, “dry” RI is reduced, and the surrounding medium is replaced with a solution that has the same RI of the delipidated tissue. The other approach consists of directly acting on the surrounding medium, immerging the brain in solutions able to increase the RI of the medium to homogenize it with the components of our tissue. In conclusion, the clearing approaches operate in two ways: on the brain components or on the surrounding medium. Depending on the clearing approach researchers use, the final RI for cleared brain or other tissues ranges from 1.33 (water RI) to 1.6 (lipid RI).

In this section, we review all of the existing clearing methods. Different classifications have been proposed (Richardson and Lichtman, 2015; Silvestri et al., 2016; Tainaka et al., 2016). Still, we try to gather them into three groups: (1) hydrophobic-reagent or organic solvent-based clearing methods, (2) hydrophilic-reagent or water-based clearing methods, and finally (3) hydrogel-based clearing methods.

It is worth emphasizing that in this review, we are focused on the brain, but clearing methods can be applied to many other organs.

### Hydrophobic-Reagent or Organic Solvent-Based Clearing Methods

More than a century ago, Spalteholz described, for the first time, a clearing approach for fixed tissue using organic solvents (Spalteholz, 1914). He replaced water with a high-RI mixture, composed of benzyl benzoate and methyl salicylate, and observed that biological tissue became almost entirely transparent. In general, hydrophobic-reagent methods involve organic solvents with high-RI and provide remarkable transparency of large samples (i.e., brain) in a quick time (1–2 days), perfect for a whole-brain pipeline. Due to their rapid “brain-transparentizing” capability, this class is the most used among clearing techniques (Costantini et al., 2019). Since tissues are mainly composed of water, the main principle of this method is based on dehydration and RI matching. The first step consists of water removal by organic solvent as alcohols or ethers, which are also able to solvate a small fraction of lipids. Dehydration usually leads to sample shrinkage and hardening whereas agents used in the second step, have the function to match the high-RI of water-free tissues and also to remove the remaining lipids.

This general scheme, composed of dehydration and RI matching, has been later used by Dodt, replacing methyl salicylate with benzyl alcohol. BABB (the acronym of agents used) has been created from this change (Dodt et al., 2007). A relevant limitation of organic solvent-based clearing methods is related to the dehydration step, which often leads to fluorescent protein quenching. For that reason, this method is not suitable for the endogenous reporters as GFP or tdTomato. Thus, using this type of clearing on transgenic or transfected mice is discouraged. The introduction of tetrahydrofuran (THF) and dibenzyl ether (DBE), respectively, as dehydrated and RI matching agent has developed 3DISCO techniques and has improved fluorescence preservation that lasts for a few days (Becker et al., 2012; Ertürk et al., 2012). This has been possible by the elimination of peroxides generating from THF and DBE before usage of these solvents. Other improvements have allowed the development of many variants of DISCO-based techniques. Renier et al. (2014) using iDISCO, overcame the problem related to fluorescence quenching, combining clearing with whole-mount immunohistochemistry, with the aim to direct an antibody against the different fluorescent proteins. Anyway, they have also used a combination of phosphate-buffer saline (PBS) and dimethyl sulfoxide (DMSO) also to preserve GFP expression for a few days. These techniques make tissue highly transparent, and they allow permanent preservation of specimens, owing to their hardening. Moreover, organic solvent-based techniques are extremely rapid. To understand their speed, just think that an entire brain is cleared in only a few days. However, the use of many toxic and dangerous agents, the scarce availability of appropriate immersion lenses for imaging, the fluorescent protein quenching, have induced researchers to develop alternative approaches.

### Hydrophilic-Reagent or Water-Based Clearing Methods

The extensive use of endogenous fluorescent reporters, as GFP or tdTomato protein, has driven the development of new clearing protocols replacing organic solvents with water-soluble reagents. These hydrophilic clearing methods exploit two different approaches: passive immersion in high-RI aqueous solution, and delipidation with hyperhydrating reagents. The former approach is based on a direct immersion of the sample in a high-RI solution to clear the sample gradually. In detail, saturated sugar solutions that are prepared with elevated concentration of sucrose or fructose are used in SeeDB and FRUIT techniques, respectively (Ke et al., 2013; Hou et al., 2015). The practical drawbacks of using high-sugar concentration are the high viscosity that limits sample manipulation and could introduce air bubbles, the potential precipitation at room temperature, and browning coloration at more elevated temperatures. The sugar viscosity causes slow penetration inside the sample, thereby extending clearing time up to months. This problem can be overcome using different water-based reagents with low viscosity as 2,2′-thiodiethanol (TDE) and FocusClear (Chiang et al., 2002; Staudt et al., 2007; Aoyagi et al., 2015; Costantini et al., 2015). The use of this latter is limited by its expensive cost.

On the other side, hyperhydrating reagents operate increasing osmotic pressure and water flux inside the cell. The water entrance tries to maintain an aqueous environment for fluorescence preservation, while the simultaneous use of detergents for lipid removal lowers the tissue RI. Hyperhydrating reagents are also used to hydrate and often partially denature proteins, the other major tissue component, further reducing the overall RI closer to that of water. The Miyawaki group has discovered the clearing ability of urea, thereby developing *Scale* approach (Hama et al., 2011). Urea is able to simultaneously penetrate and to break protein folded regions, requiring water to adjust RI. *Scale*, which involves urea, glycerol, and a detergent (Triton-X), was the first technique taking advantage of hyperhydrating reagents. In general, these protocols produce abundant hydration that leads to an optimal “specimen-transparentizing” but causes sample swelling. Therefore, in the *ScaleS* method, the substitution of glycerol with sorbitol was used to avoid the deformation and expansion of the sample (Hama et al., 2015). Although hydrophilic methods have overcome fluorescence preservation problems relative to the use of organic reagents, they require lengthy incubation times (from days to months) to clear only small portions of tissue. Starting from the ingredients of *Scale* solution, the Ueda group has developed an alternative approach called CUBIC. The aim was to clear entire organs and to accelerate clearing process without losing safety and preservation of protein function, typical of this clearing class (Susaki et al., 2014; Tainaka et al., 2014). They have screened many chemical agents and find that a series of amino alcohols have both decolorization and delipidation functions. Therefore, a mixture of selected amino alcohols, together with Triton-X and urea, has been included in the CUBIC protocol. High concentrations of Triton-X maximize lipid removal but also damage some protein epitopes. To allow whole-mount immunostaining, CUBIC protocols found an optimal concentration of detergent that permeabilizes membranes while preserving epitopes useful for antibody labeling. New CUBIC versions are extended even in the expansion microscopy (ExM) field (CUBIC-X) (Murakami et al., 2018). ExM is a method able to improve the resolution of light microscopy by physically expanding biological samples (Chen et al., 2015). This approach allows to reconstruct full details of small structures (i.e., synaptic connections). In conclusion, another strategy that exploits hyperhydrating reagents is *Clear*^*T*^ that uses a solution composed of water and formamide. Starting from *Clear*^*T*^, various methods were proposed as *Clear^*T*2^* and RTF (Kuwajima et al., 2013; Yu et al., 2018).

### Tissue Transformation-Based Clearing Methods

In the last years, new clearing approaches based on tissue transformation have been developed to combine the advantages of the abovementioned techniques. In 2013, the Deisseroth group was the first to introduce a hydrogel-based clearing method, called CLARITY (Chung et al., 2013). The basic idea behind CLARITY is to transform a biological tissue, in our case brain, into a hydrogel–tissue hybrid. The hydrogel, which is mainly composed of acrylamide monomers, has the function of stabilizing dispersed proteins and nucleic acid by covalent bonds. Moreover, this hybrid construct has to support and preserve tissue architecture after lipid removal. In general, for every tissue, lipids have a structural function, but as we have explained before, they are the primary source of scattering. Thus, their elimination facilitates achieving brain transparency. The removal of all lipids from the tissue using a high concentration of detergent [in this case, sodium dodecyl sulfate (SDS)] is a process that takes a long time, typically many weeks for an entire murine brain. To reduce the incubation time of the sample, an electrophoretic field could be applied to accelerate the diffusion of the ionic detergent.

Furthermore, the large gel meshes allow macromolecule penetration, like antibodies or fluorescent dyes, and the hydrogel itself increases the preservation of epitopes. For this reason, CLARITY can often be coupled with immunostaining techniques for the imaging of large tissue. Passive diffusion of probes requires longer incubation times, and the application of stochastic electric field has thereby sped up their diffusion (Kim S.Y. et al., 2015). During the years, many variants of CLARITY have been implemented. Researchers have looked for alternative methods in which passive diffusion of detergent has been preferred to electrophoretic transport. Also, the index-matching solution Focus Clear has been replaced with cheaper ones. Thus, PACT, PARS, CLARITY/TDE, CLARITY/glycerol are developed (Tomer et al., 2014; Yang et al., 2014; Costantini et al., 2015). Harsh conditions applied in the clearing process could cause troubles for protein antigenicity, fluorescence reporters, and tissue architecture. Chung lab has addressed these limitations by promoting two different techniques. SWITCH protects protein antigenicity for a rapid tissue clearing and unlimited rounds of antibody labeling (Murray et al., 2015), while SHIELD uses epoxides as chemical compounds able to create *intra e* intermolecular crosslinking in order to preserve fluorescence and probe-binding capability (Park et al., 2019). Moreover, these hydrogel-based clearing approaches have demonstrated useful for super resolution imaging, reconstructing details of neuronal projections, or even synaptic contacts (Ku et al., 2016). MAP technique exploits the idea of brain expansion, using a hydrogel that is isotropically expanded. However, in contrast to classic ExM, this approach avoids protein digestion, then the entire proteome is preserved. Although the elevated cost and long process, these hydrogel-based methods are commonly applied over the whole brain or other organs with much more safety than organic solvent ones.

## Quantifying Neuronal Activation Across the Entire Murine Brain

After tagging activated neurons and preparing samples for imaging, murine brains have to be reconstructed in 3D with some high-throughput optical microscope. In this section, we review imaging methods used for whole-brain activation mapping, together with the software tools necessary to extract quantitative information from raw data.

### Imaging

Three-dimensional optical imaging of biological tissue is traditionally achieved using confocal (Conchello and Lichtman, 2005) or two-photon microscopy (Zipfel et al., 2003). These methods afford three-dimensional resolution (which is also known as “optical sectioning”) either by removing out of focus fluorescence with a spatial filter (confocal microscopy) or by restricting fluorescence excitation to the focus of the objective lens (two-photon microscopy). However, standard implementations of both techniques are not suitable for whole-brain reconstruction for two reasons. First, they are point-scanning methods, meaning that the image is reconstructed point-by-point. This approach is inherently slow, with typical volumetric imaging rates in the order of 10^–4^ ÷ 10^–3^ mm^3^/s. Considering that a mouse brain is about 1 cm^3^, this means that 10 to 100 days are needed to fully reconstruct one full murine encephalon. Second, optical sectioning in two-photon or confocal microscopy is proportional to the numerical aperture (NA) of the imaging objective, i.e., the angle of emitted light that is collected by the lens. Typically, NA is inversely proportional to the objective working distance, i.e., the distance between the first lens and the focal plane. Thus, high-NA objectives, which are needed to achieve proper 3D resolution in confocal or two-photon microscopy, usually have limited working distances that cannot encompass the entire murine brain.

**FIGURE 4:**
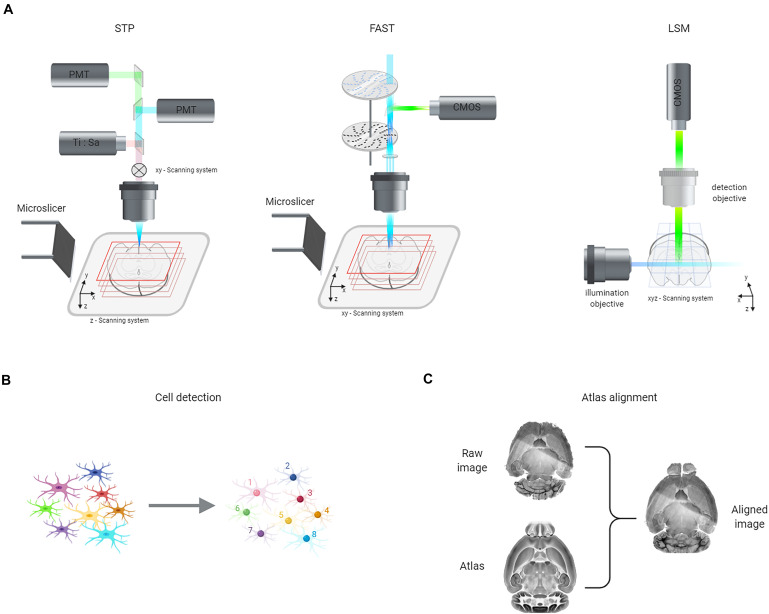
Representation of the three steps necessary to obtain neuronal quantification across the entire murine brain. **(A)** Scheme of advanced imaging approaches for whole-brain reconstruction: serial two-photon tomography (STP), block-face serial microscopy tomography (FAST), and light-sheet microscopy (LSM). **(B)** The spatial position of individual cells **(left)** can be automatically detected **(right)** using various algorithms, including machine learning ones (see text). **(C)** Finally, brain volumes can be spatially aligned to reference atlas using different computational approaches (see text).

To overcome these limitations several approaches have been developed during the years. Serial two-photon tomography (STP) incorporates a vibratome inside a standard two-photon microscope, reconstructing the volume by a continuous sequence of cutting and imaging operations (Figure 4A; Ragan et al., 2012). In this way, only the sample layers closer to the vibratome cut are imaged, allowing the use of standard high-NA objectives with limited working distance. To reduce reconstruction times, a sampling strategy is usually adopted, acquiring one optical section (1 to 2-μm thick) every 50 or 100 μm. STP has been exploited to study neuronal activation in Fos-GFP and Arc-dVenus transgenic mice (Kim Y. et al., 2015; Vousden et al., 2015).

Speed up of confocal microscopy can be obtained by parallel scanning. Spinning-disk approaches exploit multiple pinholes arranged on a rotating disk to image multiple spots simultaneously, increasing the volumetric imaging rate to 10^–1^ mm^3^/s (Wilson, 2010). Seiriki et al. (2017) developed block-face serial microscopy tomography (FAST), a spinning-disk confocal system coupled with a vibratome (Figure 4A) that can image entire mouse brains with micron resolution in 2.5 h. The authors reported the use of FAST to map fluorescent neurons in Arc-dVenus mice during acute vs. chronic restraint stress (Seiriki et al., 2017).

Sectioning methods, like FAST and STP, are, in general, used without any tissue clearing since the microscope does not need to penetrate deep inside the sample. In addition, the specimen needs to be sufficiently stiff to be adequately cut by the vibratome, a requirement incompatible with most clearing protocols. On the one hand, avoiding tissue clearing simplifies and speeds up experimental procedures. On the other hand, this prevents exogenous staining of samples, limiting the application of these methods to animal models providing intrinsic fluorescence.

The technique of choice in combination with whole-brain clearing is light-sheet microscopy (LSM) (Dodt et al., 2007; Keller and Dodt, 2012; Ueda et al., 2020). In this method, the sample is illuminated from the side with a thin sheet of light, and fluorescence is collected along an axis perpendicular to the illumination plane (Figure 4A). In this way, optical sectioning is achieved in a plane-scanning rather than a point-scanning approach. LSM is thus considerably faster than confocal or two-photon microscopy: a whole mouse brain can be reconstructed in a time ranging from hours to minutes, depending on the resolution. Indeed, a key advantage of LSM is its flexibility: the resolution of the system can be modulated from tens of microns—sufficient to discriminate cell bodies when neuronal processes are not labeled—to less than 1 μm—allowing distinguishing axons and dendrites. Low-resolution LSM has the merits of a simple optical layout and the reasonable size of the generated datasets; for these reasons, several groups were able to use it to study neuronal activation in large behavioral cohorts (Susaki et al., 2014; Renier et al., 2016; Tatsuki et al., 2016; Ye et al., 2016). However, when labeling of active neurons includes small processes, a subcellular resolution is needed to distinguish cell bodies from bundles of axons or dendrites. For instance, Ye et al. (2016) used low-resolution LSM on TRAP mice and were forced to exclude several brain regions from their analysis. High-resolution LSM can instead produce a quantitative mapping of neuronal activation independently of the labeling strategy used. The challenges, in this case, are represented by the size of the datasets produced (usually exceeding one TeraByte per brain) and the need for more complex optical systems. Indeed, high-resolution imaging through several millimeters of tissue introduces optical artifacts even with the best possible clearing. For instance, specimen-induced defocus needs to be corrected automatically to produce sharp images (Tomer et al., 2014; Silvestri et al., 2017; Matsumoto et al., 2019). Another issue that has to be addressed is the presence of shadowing artifacts introduced by adsorbing or scattering objects in the sample. Different methods have been proposed in this respect, including the use of non-Gaussian laser beams (Fahrbach and Rohrbach, 2012; Müllenbroich et al., 2018a,b) and axial sweeping of the excitation light sheet (Chakraborty et al., 2019; Voigt et al., 2019). Anyhow, whatever optical improvement must rely on a good optical clearing, which is an essential prerequisite for the use of high-resolution LSM. The group of Hiroki Ueda has pioneered IEG mapping at subcellular resolution, demonstrating the potential of this approach to quantify neuronal activation across the entire murine brains without excluding any areas (Murakami et al., 2018; Matsumoto et al., 2019; Susaki et al., 2020).

Whatever the microscopy method used to image brain samples, the next step is to transform the raw images—which are just a matrix of gray values—into semantically relevant information. This process involves two different phases that could be performed in parallel: cell detection (Figure 4B) and atlas registration (Figure 4C).

### Cell Detection

Automatic detection or segmentation of labeled cells is a well-known problem in biomedical image analysis, and many different methods have been developed during the years (Acciai et al., 2016; Magliaro et al., 2019). However, whole-brain images present some peculiar challenges that need to be faced. First, datasets are usually extensive, ranging from tens of gigabytes to tens of terabytes for a single sample. Thus, algorithms must be fast and scalable. Second, in LSM images, the contrast is very heterogeneous between deep and superficial brain regions since excitation and fluorescence cross variable thicknesses of biological tissue. Third, when the entire neuron is filled with the fluorescent label (e.g., when using transgenic strategies), an additional problem is represented by the presence of bright axons or dendrites, which may confound the detection algorithm.

Standard pipelines for cell detection use a combination of filters to homogenize contrast and highlight spherical objects, followed by adaptive thresholding of the images and then some operations/filters on binary data to refine segmentation. Such pipelines are highly parametrical: estimation of parameters is usually done on a small training set of manually annotated images. Sometimes, different sets of parameters are estimated for different brain areas to improve accuracy (Seiriki et al., 2017). Standard image processing pipelines are effective when labeling is confined to cell bodies, as in anti-c-fos immunohistochemistry (Renier et al., 2016), or when imaging quality is highly homogeneous, as in STP (Ragan et al., 2012) or FAST (Seiriki et al., 2017). Their use in LSM images of transgenic animals has been sometimes reported, but on a subset of brain regions (Ye et al., 2016), or on the whole brain but without a clear evaluation of the accuracy of the results (Menegas et al., 2015; DeNardo et al., 2019).

Machine learning approaches can be used to cope with complex or inhomogeneous images. In these methods, a model for classification of pixels or image transformation is trained using example data (“ground truth”) provided by the user. As a general principle, the performances of the model increase with its complexity (the number of hidden parameters). However, more complex models require more ground truth for successful training. Thus, in practice, a trade-off between performances and manual annotation has to be found.

Ilastik (Berg et al., 2019) is a popular image-processing tool implementing simpler models—a random forest classifier based on a set of user-defined image features. This software allows real-time training and testing, together with a user-friendly environment suitable also for researchers with limited background in computer science. Menegas and coworkers reported its use in whole-brain LSM images, although not for an application related to IEG mapping (Menegas et al., 2015).

More sophisticated models, like the multilayered neural network used in the emerging field of deep learning (Gupta et al., 2019), have the potential to process images with human-level (or even super-human) performances. They are an established standard in the analysis of natural images, and their application to whole-brain image analysis has been reported (Kim Y. et al., 2015; Kirst et al., 2020; Todorov et al., 2020). Even if they are extremely powerful, their use is still quite limited in the field, probably because they need large human-annotated training datasets. In this respect, strategies to speed up labeling, e.g., by pinpointing the position of the neuronal soma rather than segmenting the neuronal volume, are promising to generate annotations much faster (Frasconi et al., 2014; Silvestri et al., 2015).

### Spatial Registration to Reference Atlas

Detected cells must then be assigned to a specific brain region to allow precise quantification of which areas are elicited during a specific behavior. Although anatomy experts can directly draw major regions on the collected images (Seiriki et al., 2017), the standard choice is to refer to standard atlases, like the classic Franklin and Paxinos (Paxinos and Franklin, 2004) or the more recent one from the Allen Institute for Brain Science (Jones et al., 2009). This latter is the average of more than 1,000 whole-brain images obtained with STP and is associated with a 3D parcelation operated by a group of expert neuroanatomist. By registering, i.e., aligning, a sample image to the atlas template (or vice versa), detected cells can be directly assigned to a specific brain region.

Image registration is performed by finding the best transformation mapping one image into the other and is thus defined by the transformation itself, a quality metric, and an optimization strategy. Intersample differences are usually quite significant because of both biological variability (Scholz et al., 2016) and the deformation introduced by chemical clearing (Kutten et al., 2016). Global affine transformations, which are composed of translation, rotation, global (anisotropic) scaling, and shear, are usually not enough to match samples and reference. Conversely, non-linear local transformations, like B-spline (Klein et al., 2010; Fürth et al., 2018) or symmetric diffeomorphisms (Avants et al., 2011; Kutten et al., 2016), can recover sample deformations and provide reliable mapping onto the atlas.

The parameters of any transformation are obtained by maximizing some measures of registration quality. The most commonly used are cross-correlation—which works nicely for images sharing the same type of labeling—and mutual information—which performs well when the datasets are based on different stains. By coupling the quality metrics of choice with a suitable optimization algorithm, it is then possible to find the best transformation, mapping the sample to the atlas or vice versa. The most common 3D registration tools used in the field are probably Elastix (Klein et al., 2010) and ANTs (Advanced Normalization Tools) (Avants et al., 2011), and they have also been incorporated in larger projects like ClearMap (Renier et al., 2016) or CUBIC-X (Murakami et al., 2018).

In practice, registration is performed on images at coarse resolution, typically 25-μm-pixel size or worse. To facilitate the process, a reference channel containing either tissue autofluorescence (Kim Y. et al., 2015; Menegas et al., 2015; Renier et al., 2016; Ye et al., 2016) or some kind of nuclear staining [e.g., propidium iodide (Murakami et al., 2018)] is used rather than the channel related to labeled cells. Several authors also suggested to first perform a mutual registration of all the samples into an “average brain,” followed by semi-manual registration of this latter to the reference atlas (Vousden et al., 2015; Ye et al., 2016; Murakami et al., 2018). Finally, it is worth noting that direct 3D registration is often quite challenging, especially for cleared samples that underwent severe deformations. Some groups proposed hybrid strategies, where a first 3D coarse alignment is followed by 2D accurate registration slice-by-slice (Tainaka et al., 2014; Fürth et al., 2018; Murakami et al., 2018).

## Conclusion and Outlook

It is not yet clear how neuronal activity is correlated to specific behaviors. Stimuli from the world outside activate different neuronal pathways inside the brain. In turn, this activation triggers a cascade of events that eventually result in a precise behavior. Whole-brain mapping is an emerging technique to understand how the brain drives specific behaviors, even though it is still rarely used because of its multidisciplinary nature. Indeed, this sector of neuroscience ranges from genetics, sample preparation to imaging, and image analysis. This paper reviews the latest developments of each field with the general aim to combine every area of interest into a single pipeline for a routine and large-scale use.

**FIGURE 5:**
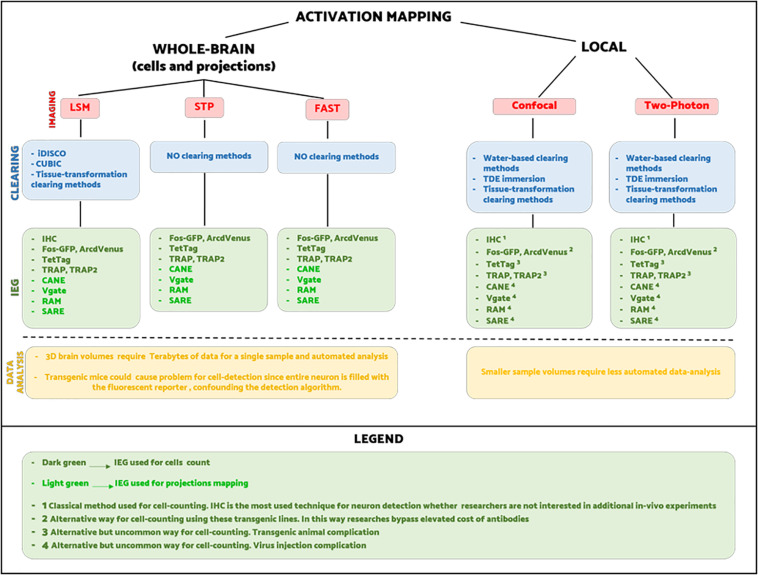
Schematic of experimental pipelines that could be used for activation mapping.

Nowadays, there is not a unique way for visualizing neuronal activation on a brain-wide scale. Hence, it is up to researchers to choose the combination of methods best suited to their experimental purpose. In Figure 5, we try to summarize all the different experimental pipelines for whole-brain activity mapping. As the scheme shows, the number of possible combinations between labeling, clearing, imaging, and image processing is very high, and thus the choice falls on practical aspects. For instance, regarding short-term experiments where animals are sacrificed immediately after the behavioral task, it is preferable to use classic techniques as IHC rather than viruses or transgenes. On the other hand, transgenic mice are more appropriate for experiments that need to last over time, allowing multiple behavioral tests.

Whole-brain activation mapping naturally complements anatomical mapping, both in terms of cellular architecture (Kim et al., 2017) and of long-range axonal projections (Oh et al., 2014; Economo et al., 2016; Winnubst et al., 2019). Indeed, the knowledge of structural wiring of neuronal networks helps the interpretation of activity data. For example, co-activation of two brain areas could occur either because they are directly connected or via the involvement of a third brain region. This kind of information cannot be inferred from activation data only, but needs anatomical investigation and targeted stimulation with opto- or chemogenetics (Ye et al., 2016; Franklin et al., 2017; Vetere et al., 2017).

The majority of the techniques discussed in this review have been hitherto applied only to rodents, mainly mice. An important step for future research would be to map behavior-related neuronal activation in larger mammalian species. While transgenic strategies are currently not applicable to non-human primates (NHP), viral approaches like RAM could be, in principle, switched to map-activated neurons in different animals (Sørensen et al., 2016). Indeed, large-scale clearing and imaging methods have been demonstrated in small NHP like the marmoset (Seiriki et al., 2017; Susaki et al., 2020). When coupled with standard neuroimaging like functional magnetic resonance imaging (fMRI) or positron-emission tomography (PET), single-cell activation mapping in NHP could provide a unique framework to better understand the relationship between neuronal activity and imaging data. In turn, this would improve the interpretation of fMRI and PET in humans, with relevant implications for clinical practices.

In conclusion, this review confirms that the brain mapping is a constantly evolving field of neuroscience, and we are convinced that these approaches could not only be used in a routine way but also and overall, on a larger scale, in the near future.

## Author Contributions

All authors listed have made a substantial, direct and intellectual contribution to the work, and approved it for publication.

## Conflict of Interest

The authors declare that the research was conducted in the absence of any commercial or financial relationships that could be construed as a potential conflict of interest.

## References

[B1] Acciai L. Soda P. Iannello G. (2016). Automated neuron tracing methods: an updated account. *Neuroinformatics* 14 353–367. 10.1007/s12021-016-9310-0 27447185

[B2] Aharoni D. Khakh B. S. Silva A. J. Golshani P. (2019). All the light that we can see: a new era in miniaturized microscopy. *Nat. Methods* 16 11–13. 10.1038/s41592-018-0266-x 30573833 PMC8320687

[B3] Ahrens M. B. Orger M. B. Robson D. N. Li J. M. Keller P. J. (2013). Whole-brain functional imaging at cellular resolution using light-sheet microscopy. *Nat. Methods* 10 413–420. 10.1038/nmeth.2434 23524393

[B4] Aoyagi Y. Kawakami R. Osanai H. Hibi T. Nemoto T. (2015). A rapid optical clearing protocol using 2,2’-thiodiethanol for microscopic observation of fixed mouse brain. *PLoS One* 10:e0116280. 10.1371/journal.pone.0116280 25633541 PMC4310605

[B5] Avants B. B. Tustison N. J. Song G. Cook P. A. Klein A. Gee J. C. (2011). A reproducible evaluation of ANTs similarity metric performance in brain image registration. *NeuroImage* 54 2033–2044. 10.1016/j.neuroimage.2010.09.025 20851191 PMC3065962

[B6] Bahrami S. Drabløs F. (2016). Gene regulation in the immediate-early response process. *Adv. Biol. Regul.* 62 37–49. 10.1016/j.jbior.2016.05.001 27220739

[B7] Bartel D. P. Sheng M. Lau L. F. Greenberg M. E. (1989). Growth factors and membrane depolarization activate distinct programs of early response gene expression: dissociation of fos and jun induction. *Genes Dev.* 3 304–313. 10.1101/gad.3.3.304 2498163

[B8] Barth A. L. Gerkin R. C. Dean K. L. (2004). Alteration of neuronal firing properties after in vivo experience in a FosGFP transgenic mouse. *J. Neurosci.* 24 6466–6475. 10.1523/JNEUROSCI.4737-03.2004 15269256 PMC6729874

[B9] Becker K. Jährling N. Saghafi S. Weiler R. Dodt H. U. (2012). Chemical clearing and dehydration of GFP expressing mouse brains. *PLoS One* 7:e33916. 10.1371/journal.pone.0033916 22479475 PMC3316521

[B10] Berg S. Kutra D. Kroeger T. Straehle C. N. Kausler B. X. Haubold C. (2019). ilastik: interactive machine learning for (bio)image analysis. *Nat. Methods* 16 1226–1232. 10.1038/s41592-019-0582-9 31570887

[B11] Carrillo-Reid L. Yang W. Kang Miller J. Peterka D. S. Yuste R. (2017). Imaging and optically manipulating neuronal ensembles. *Annu. Rev. Biophys.* 46 271–293. 10.1146/annurev-biophys-070816-033647 28301770

[B12] Chakraborty T. Driscoll M. K. Jeffery E. Murphy M. M. Roudot P. Chang B. J. (2019). Light-sheet microscopy of cleared tissues with isotropic, subcellular resolution. *Nat. Methods* 16 1109–1113. 10.1038/s41592-019-0615-4 31673159 PMC6924633

[B13] Chen F. Tillberg P. W. Boyden E. S. (2015). Expansion microscopy. *Science* 347 543–548. 10.1126/science.1260088 25592419 PMC4312537

[B14] Chiang A. S. Lin W. Y. Liu H. P. Pszczolkowski M. A. Fu T. F. Chiu S. L. (2002). Insect NMDA receptors mediate juvenile hormone biosynthesis. *Proc. Natl. Acad. Sci. U.S.A.* 99 37–42. 10.1073/pnas.012318899 11773617 PMC117510

[B15] Chung K. Wallace J. Kim S. Y. Kalyanasundaram S. Andalman A. S. Davidson T. J. (2013). Structural and molecular interrogation of intact biological systems. *Nature* 497 332–337. 10.1038/nature12107 23575631 PMC4092167

[B16] Cole A. J. Saffen D. W. Baraban J. M. Worley P. F. (1989). Rapid increase of an immediate early gene messenger RNA in hippocampal neurons by synaptic NMDA receptor activation. *Nature* 340 474–476. 10.1038/340474a0 2547165

[B17] Conchello J. A. Lichtman J. W. (2005). Optical sectioning microscopy. *Nat. Methods* 2 920–931. 10.1038/nmeth815 16299477

[B18] Costantini I. Cicchi R. Silvestri L. Vanzi F. Pavone F. S. (2019). In-vivo and ex-vivo optical clearing methods for biological tissues: review. *Biomed. Opt. Express* 10 5251–5267. 10.1364/boe.10.005251 31646045 PMC6788593

[B19] Costantini I. Ghobril J. P. di Giovanna A. P. Allegra Mascaro A. L. Silvestri L. Müllenbroich M. C. (2015). A versatile clearing agent for multi-modal brain imaging. *Sci. Rep.* 5 1–9. 10.1038/srep09808 25950610 PMC4423470

[B20] Cowansage K. K. Shuman T. Dillingham B. C. Chang A. Golshani P. Mayford M. (2014). Direct reactivation of a coherent neocortical memory of context. *Neuron* 84 432–441. 10.1016/j.neuron.2014.09.022 25308330 PMC4372249

[B21] DeNardo L. Luo L. (2017). Genetic strategies to access activated neurons. *Curr. Opin. Neurobiol.* 45 121–129. 10.1016/j.conb.2017.05.014 28577429 PMC5810937

[B22] DeNardo L. A. Liu C. D. Allen W. E. Adams E. L. Friedmann D. Fu L. (2019). Temporal evolution of cortical ensembles promoting remote memory retrieval. *Nat. Neurosci.* 22 460–469. 10.1038/s41593-018-0318-7 30692687 PMC6387639

[B23] Dodt H. U. Leischner U. Schierloh A. Jährling N. Mauch C. P. Deininger K. (2007). Ultramicroscopy: three-dimensional visualization of neuronal networks in the whole mouse brain. *Nat. Methods* 4 331–336. 10.1038/nmeth1036 17384643

[B24] Dombeck D. A. Harvey C. D. Tian L. Looger L. L. Tank D. W. (2010). Functional imaging of hippocampal place cells at cellular resolution during virtual navigation. *Nat. Neurosci.* 13 1433–1440. 10.1038/nn.2648 20890294 PMC2967725

[B25] Economo M. N. Clack N. G. Lavis L. D. Gerfen C. R. Svoboda K. Myers E. W. (2016). A platform for brain-wide imaging and reconstruction of individual neurons. *eLife* 5:e10566. 10.7554/eLife.10566 26796534 PMC4739768

[B26] Ertürk A. Becker K. Jährling N. Mauch C. P. Hojer C. D. Egen J. G. (2012). Three-dimensional imaging of solvent-cleared organs using 3DISCO. *Nat. Protoc.* 7 1983–1995. 10.1038/nprot.2012.119 23060243

[B27] Fahrbach F. O. Rohrbach A. (2012). Propagation stability of self-reconstructing Bessel beams enables contrast-enhanced imaging in thick media. *Nat. Commun.* 3:632. 10.1038/ncomms1646 22252556

[B28] Feil R. Wagner J. Metzger D. Chambon P. (1997). Regulation of Cre recombinase activity by mutated estrogen receptor ligand-binding domains. *Biochem. Biophys. Res. Commun.* 237 752–757. 10.1006/bbrc.1997.7124 9299439

[B29] Fosque B. F. Sun Y. Dana H. Yang C. T. Ohyama T. Tadross M. R. (2015). Labeling of active neural circuits in vivo with designed calcium integrators. *Science* 347 755–760. 10.1126/science.1260922 25678659

[B30] Fowler T. Sen R. Roy A. L. (2011). Regulation of primary response genes. *Mol. Cell* 44 348–360. 10.1016/j.molcel.2011.09.014 22055182 PMC3212756

[B31] Franklin T. B. Silva B. A. Perova Z. Marrone L. Masferrer M. E. Zhan Y. (2017). Prefrontal cortical control of a brainstem social behavior circuit. *Nat. Neurosci.* 20 260–270. 10.1038/nn.4470 28067904 PMC5580810

[B32] Frasconi P. Silvestri L. Soda P. Cortini R. Pavone F. S. Iannello G. (2014). Large-scale automated identification of mouse brain cells in confocal light sheet microscopy images. *Bioinformatics* 30 i587–i593. 10.1093/bioinformatics/btu469 25161251 PMC4147922

[B33] Fürth D. Vaissière T. Tzortzi O. Xuan Y. Märtin A. Lazaridis I. (2018). An interactive framework for whole-brain maps at cellular resolution. *Nat. Neurosci.* 21 139–153. 10.1038/s41593-017-0027-7 29203898 PMC5994773

[B34] Garner A. R. Rowland D. C. Hwang S. Y. Baumgaertel K. Roth B. L. Kentros C. (2012). Generation of a synthetic memory trace. *Science* 335 1513–1516. 10.1126/science.1214985 22442487 PMC3956300

[B35] Ghosh A. Ginty D. D. Bading H. Greenberg M. E. (1994). Calcium regulation of gene expression in neuronal cells. *J. Neurobiol.* 25 294–303. 10.1002/neu.480250309 7910846

[B36] Girasole A. E. Lum M. Y. Nathaniel D. Bair-Marshall C. J. Guenthner C. J. Luo L. (2018). A subpopulation of striatal neurons mediates levodopa-induced dyskinesia. *Neuron* 97 787–795.e6. 10.1016/j.neuron.2018.01.017 29398356 PMC6233726

[B37] Greenberg M. E. Ziff E. B. Greene L. A. (1986). Stimulation of neuronal acetylcholine receptors induces rapid gene transcription. *Science* 234 80–83. 10.1126/science.3749894 3749894

[B38] Guenthner C. J. Miyamichi K. Yang H. H. Heller H. C. Luo L. (2013). Permanent genetic access to transiently active neurons via TRAP: targeted recombination in active populations. *Neuron* 78 773–784. 10.1016/j.neuron.2013.03.025 23764283 PMC3782391

[B39] Gupta A. Harrison P. J. Wieslander H. Pielawski N. Kartasalo K. Partel G. (2019). Deep learning in image cytometry: a review. *Cytometry Part A* 95 366–380. 10.1002/cyto.a.23701 30565841 PMC6590257

[B40] Guzowski J. F. Worley P. F. (2001). Cellular compartment analysis of temporal activity by fluorescence in situ hybridization (catFISH). *Curr. Protoc. Neurosci.* 15 1.8.1–1.8.16. 10.1002/0471142301.ns0108s15 18428454

[B41] Hama H. Hioki H. Namiki K. Hoshida T. Kurokawa H. Ishidate F. (2015). ScaleS: an optical clearing palette for biological imaging. *Nat. Neurosci.* 18 1518–1529. 10.1038/nn.4107 26368944

[B42] Hama H. Kurokawa H. Kawano H. Ando R. Shimogori T. Noda H. (2011). Scale: a chemical approach for fluorescence imaging and reconstruction of transparent mouse brain. *Nat. Neurosci.* 14 1481–1488. 10.1038/nn.2928 21878933

[B43] Hasan M. T. Althammer F. Silva da Gouveia M. Goyon S. Eliava M. Lefevre A. (2019). A fear memory engram and its plasticity in the hypothalamic oxytocin system. *Neuron* 103 133–146.e8. 10.1016/j.neuron.2019.04.029 31104950

[B44] He Q. Wang J. Hu H. (2019). Illuminating the activated brain: emerging activity-dependent tools to capture and control functional neural circuits. *Neurosci. Bull.* 35 369–377. 10.1007/s12264-018-0291-x 30255458 PMC6527722

[B45] Helmchen F. Denk W. (2005). Deep tissue two-photon microscopy. *Nat. Methods* 2 932–940. 10.1038/nmeth818 16299478

[B46] Hou B. Zhang D. Zhao S. Wei M. Yang Z. Wang S. (2015). Scalable and DiI-compatible optical clearance of the mammalian brain. *Front. Neuroanat.* 9:19. 10.3389/fnana.2015.00019 25759641 PMC4338786

[B47] Hughes P. Lawlor P. Dragunow M. (1992). Basal expression of Fos, Fos-related, Jun, and Krox 24 proteins in rat hippocampus. *Mol. Brain Res.* 13 355–357. 10.1016/0169-328X(92)90219-2 1320724

[B48] Hunt S. P. Pini A. Evan G. (1988). Induction of c-fos-like protein in spinal cord neurons following sensory stimulation. *Nature* 328 632–634. 10.1038/328632a0 3112583

[B49] Ishii K. K. Osakada T. Mori H. Miyasaka N. Yoshihara Y. Miyamichi K. (2017). A labeled-line neural circuit for pheromone-mediated sexual behaviors in mice. *Neuron* 95 123–137.e8. 10.1016/j.neuron.2017.05.038 28648498

[B50] Jacques S. L. (2013). Optical properties of biological tissues: a review. *Phys. Med. Biol.* 58 R37–R61. 10.1088/0031-9155/58/11/R37 23666068

[B51] Jiang-Xie L. F. Yin L. Zhao S. Prevosto V. Han B. X. Dzirasa K. (2019). A common neuroendocrine substrate for diverse general anesthetics and sleep. *Neuron* 102 1053–1065.e4. 10.1016/j.neuron.2019.03.033 31006556 PMC6554048

[B52] Jones A. R. Overly C. C. Sunkin S. M. (2009). The allen brain atlas: 5 years and beyond. *Nat. Rev. Neurosci.* 10 821–828. 10.1038/nrn2722 19826436

[B53] Kaczmarek L. Nikolajew E. (1990). C-fos protooncogene expression and neuronal plasticity. *Acta Neurobiol. Exp.* 50 173–179. 2130639

[B54] Kawashima T. Kitamura K. Suzuki K. Nonaka M. Kamijo S. Takemoto-Kimura S. (2013). Functional labeling of neurons and their projections using the synthetic activity-dependent promoter E-SARE. *Nat. Methods* 10 889–895. 10.1038/nmeth.2559 23852453

[B55] Kawashima T. Okuno H. Nonaka M. Adachi-Morishima A. Kyo N. Okamura M. (2009). Synaptic activity-responsive element in the Arc/Arg3.1 promoter essential for synapse-to-nucleus signaling in activated neurons. *Proc. Natl. Acad. Sci. U.S.A.* 106 316–321. 10.1073/pnas.0806518106 19116276 PMC2629236

[B56] Ke M. T. Fujimoto S. Imai T. (2013). SeeDB: a simple and morphology-preserving optical clearing agent for neuronal circuit reconstruction. *Nat. Neurosci.* 16 1154–1161. 10.1038/nn.3447 23792946

[B57] Keller P. J. Dodt H. U. (2012). Light sheet microscopy of living or cleared specimens. *Curr. Opin. Neurobiol.* 22 138–143. 10.1016/j.conb.2011.08.003 21925871

[B58] Kim S. Y. Cho J. H. Murray E. Bakh N. Choi H. Ohn K. (2015). Stochastic electrotransport selectively enhances the transport of highly electromobile molecules. *Proc. Natl. Acad. Sci. U.S.A.* 112 E6274–E6283. 10.1073/pnas.1510133112 26578787 PMC4655572

[B59] Kim W. B. Cho J. H. (2017). Encoding of discriminative fear memory by input-specific LTP in the amygdala. *Neuron* 95 1129–1146.e5. 10.1016/j.neuron.2017.08.004 28823727

[B60] Kim Y. Venkataraju K. U. Pradhan K. Mende C. Taranda J. Turaga S. C. (2015). Mapping social behavior-induced brain activation at cellular resolution in the mouse. *Cell Rep.* 10 292–305. 10.1016/j.celrep.2014.12.014 25558063 PMC4294964

[B61] Kim Y. Yang G. R. Pradhan K. Venkataraju K. U. Bota M. García del Molino L. C. (2017). Brain-wide maps reveal stereotyped cell-type-based cortical architecture and subcortical sexual dimorphism. *Cell* 171 456–469.e22. 10.1016/j.cell.2017.09.020 28985566 PMC5870827

[B62] Kirst C. Skriabine S. Vieites-Prado A. Topilko T. Bertin P. Gerschenfeld G. (2020). Mapping the fine-scale organization and plasticity of the brain vasculature. *Cell* 180 780–795.e25. 10.1016/j.cell.2020.01.028 32059781

[B63] Klein S. Staring M. Murphy K. Viergever M. A. Pluim J. P. W. (2010). elastix: a toolbox for intensity-based medical image registration. *IEEE Trans. Med. Imaging* 29 196–205. 10.1109/TMI.2009.2035616 19923044

[B64] Koya E. Golden S. A. Harvey B. K. Guez-Barber D. H. Berkow A. Simmons D. E. (2009). Targeted disruption of cocaine-activated nucleus accumbens neurons prevents context-specific sensitization. *Nat. Neurosci.* 12 1069–1073. 10.1038/nn.2364 19620976 PMC2752202

[B65] Ku T. Swaney J. Park J. Y. Albanese A. Murray E. Hun Cho J. (2016). Multiplexed and scalable super-resolution imaging of three-dimensional protein localization in size-adjustable tissues. *Nat. Biotechnol.* 34 973–981. 10.1038/nbt.3641 27454740 PMC5070610

[B66] Kutten K. S. Charon N. Miller M. I. Ratnanather J. T. Matelsky J. Baden A. D. (2017). “A large deformation diffeomorphic approach to registration of CLARITY images via mutual information,” in *International Conference on Medical Image Computing and Computer-Assisted Intervention* (Berlin: Springer), 275–282. Available online at: https://link.springer.com/chapter/10.1007%2F978-3-319-66182-7_32

[B67] Kuwajima T. Sitko A. A. Bhansali P. Jurgens C. Guido W. Mason C. (2013). ClearT: a detergent- and solvent-free clearing method for neuronal and non-neuronal tissue. *Development* 140 1364–1368. 10.1242/dev.091844 23444362 PMC3912244

[B68] Lee D. Hyun J. H. Jung K. Hannan P. Kwon H. B. (2017). A calcium- A nd light-gated switch to induce gene expression in activated neurons. *Nat. Biotechnol.* 35 858–863. 10.1038/nbt.3902 28650460

[B69] Liu X. Ramirez S. Pang P. T. Puryear C. B. Govindarajan A. Deisseroth K. (2012). Optogenetic stimulation of a hippocampal engram activates fear memory recall. *Nature* 484 381–385. 10.1038/nature11028 22441246 PMC3331914

[B70] Logothetis N. K. (2008). What we can do and what we cannot do with fMRI. *Nature* 453 869–878. 10.1038/nature06976 18548064

[B71] Magliaro C. Callara A. L. Vanello N. Ahluwalia A. (2019). Gotta Trace ‘em All: a mini-review on tools and procedures for segmenting single neurons toward deciphering the structural connectome. *Front. Bioeng. Biotechnol.* 7:202. 10.3389/fbioe.2019.00202 31555642 PMC6727034

[B72] Matsumoto K. Mitani T. T. Horiguchi S. A. Kaneshiro J. Murakami T. C. Mano T. (2019). Advanced CUBIC tissue clearing for whole-organ cell profiling. *Nat. Protoc.* 14 3506–3537. 10.1038/s41596-019-0240-9 31748753

[B73] Mayford M. Reijmers L. (2016). Exploring memory representations with activity-based genetics. *Cold Spring Harb. Perspect. Biol.* 8:a021832. 10.1101/cshperspect.a021832 26684182 PMC4772104

[B74] Menegas W. Bergan J. F. Ogawa S. K. Isogai Y. Umadevi Venkataraju K. Osten P. (2015). Dopamine neurons projecting to the posterior striatum form an anatomically distinct subclass. *eLife* 4:e10032 10.7554/eLife.10032.001 PMC4598831 26322384

[B75] Michel C. M. Brunet D. (2019). EEG source imaging: a practical review of the analysis steps. *Front. Neurol.* 10:325. 10.3389/fneur.2019.00325 31019487 PMC6458265

[B76] Morgan J. I. Cohen D. R. Hempstead J. L. Curran T. (1987). Mapping patterns of c-fos expression in the central nervous system after seizure. *Science* 237 192–197. 10.1126/science.3037702 3037702

[B77] Müllenbroich M. C. Silvestri L. di Giovanna A. P. Mazzamuto G. Costantini I. Sacconi L. (2018a). High-fidelity imaging in brain-wide structural studies using light-sheet microscopy. *eNeuro* 5 1–13. 10.1523/ENEURO.0124-18.2018 30627630 PMC6325532

[B78] Müllenbroich M. C. Turrini L. Silvestri L. Alterini T. Gheisari A. Vanzi F. (2018b). Bessel beam illumination reduces random and systematic errors in quantitative functional studies using light-sheet microscopy. *Front. Cell. Neurosci.* 12:315. 10.3389/fncel.2018.00315 30294262 PMC6158350

[B79] Murakami T. C. Mano T. Saikawa S. Horiguchi S. A. Shigeta D. Baba K. (2018). A three-dimensional single-cell-resolution whole-brain atlas using CUBIC-X expansion microscopy and tissue clearing. *Nat. Neurosci.* 21 625–637. 10.1038/s41593-018-0109-1 29507408

[B80] Murray E. Cho J. H. Goodwin D. Ku T. Swaney J. Kim S. Y. (2015). Simple, scalable proteomic imaging for high-dimensional profiling of intact systems. *Cell* 163 1500–1514. 10.1016/j.cell.2015.11.025 26638076 PMC5275966

[B81] Nöbauer T. Skocek O. Pernía-Andrade A. J. Weilguny L. Martínez Traub F. Molodtsov M. I. (2017). Video rate volumetric Ca2+ imaging across cortex using seeded iterative demixing (SID) microscopy. *Nat. Methods* 14 811–818. 10.1038/nmeth.4341 28650477

[B82] Oh S. W. Harris J. a Ng L. Winslow B. Cain N. Mihalas S. (2014). A mesoscale connectome of the mouse brain. *Nature* 508 207–214. 10.1038/nature13186 24695228 PMC5102064

[B83] Park Y. G. Sohn C. H. Chen R. McCue M. Yun D. H. Drummond G. T. (2019). Protection of tissue physicochemical properties using polyfunctional crosslinkers. *Nat. Biotechnol.* 37:73. 10.1038/nbt.4281 30556815 PMC6579717

[B84] Paxinos G. Franklin K. (2004). *The Mouse Brain in Stereotaxic Coordinates.* Available online at: https://books.google.com/books?hl=it&lr=&id=EHy1QN1xv0gC&oi=fnd&pg=PR9&ots=80XUsTGL_o&sig=LH9tOACXe-6e_bS9RGg87Ka688g (accessed June 1, 2020).

[B85] Peng H. Zhou J. Zhou Z. Bria A. Li Y. Kleissas D. M. (2016). Bioimage informatics for big data. *Adv. Anat. Embryol. Cell Biol.* 219 263–272. 10.1007/978-3-319-28549-8_10 27207370

[B86] Perin R. Markram H. (2013). A computer-assisted multi-electrode patch-clamp system. *J. Vis. Exp.* 80:e50630. 10.3791/50630 24192529 PMC3948366

[B87] Pinaud R. (2004). Experience-dependent immediate early gene expression in the adult central nervous system: evidence from enriched-environment studies. *Int. J. Neurosci.* 114 321–333. 10.1080/00207450490264142 14754658

[B88] Power R. M. Huisken J. (2017). A guide to light-sheet fluorescence microscopy for multiscale imaging. *Nat. Methods* 14 360–373. 10.1038/nmeth.4224 28362435

[B89] Prevedel R. Yoon Y. G. Hoffmann M. Pak N. Wetzstein G. Kato S. (2014). Simultaneous whole-animal 3D imaging of neuronal activity using light-field microscopy. *Nat. Methods* 11 727–730. 10.1038/nmeth.2964 24836920 PMC4100252

[B90] Ragan T. Kadiri L. R. Venkataraju K. U. Bahlmann K. Sutin J. Taranda J. (2012). Serial two-photon tomography for automated ex vivo mouse brain imaging. *Nat. Methods* 9 255–258. 10.1038/nmeth.1854 22245809 PMC3297424

[B91] Ramirez S. Liu X. Lin P. A. Suh J. Pignatelli M. Redondo R. L. (2013). Creating a false memory in the hippocampus. *Science* 341 387–391. 10.1126/science.1239073 23888038

[B92] Ramirez S. Liu X. MacDonald C. J. Moffa A. Zhou J. Redondo R. L. (2015). Activating positive memory engrams suppresses depression-like behaviour. *Nature* 522 335–339. 10.1038/nature14514 26085274 PMC5583720

[B93] Redondo R. L. Kim J. Arons A. L. Ramirez S. Liu X. Tonegawa S. (2014). Bidirectional switch of the valence associated with a hippocampal contextual memory engram. *Nature* 513 426–430. 10.1038/nature13725 25162525 PMC4169316

[B94] Reijmers L. G. Perkins B. L. Matsuo N. Mayford M. (2007). Localization of a stable neural correlate of associative memory. *Science* 317 1230–1233. 10.1126/science.1143839 17761885

[B95] Renier N. Adams E. L. Kirst C. Wu Z. Azevedo R. Kohl J. (2016). Mapping of brain activity by automated volume analysis of immediate early genes. *Cell* 165 1789–1802. 10.1016/j.cell.2016.05.007 27238021 PMC4912438

[B96] Renier N. Wu Z. Simon D. J. Yang J. Ariel P. Tessier-Lavigne M. (2014). IDISCO: a simple, rapid method to immunolabel large tissue samples for volume imaging. *Cell* 159 896–910. 10.1016/j.cell.2014.10.010 25417164

[B97] Richardson D. S. Lichtman J. W. (2015). Clarifying tissue clearing. *Cell* 162 246–257. 10.1016/j.cell.2015.06.067 26186186 PMC4537058

[B98] Rodriguez E. Sakurai K. Xu J. Chen Y. Toda K. Zhao S. (2017). A craniofacial-specific monosynaptic circuit enables heightened affective pain. *Nat. Neurosci.* 20 1734–1743. 10.1038/s41593-017-0012-1 29184209 PMC5819335

[B99] Sagar S. M. Sharp F. R. Curran T. (1988). Expression of c-fos protein in brain: metabolic mapping at the cellular level. *Science* 240 1328–1331. 10.1126/science.3131879 3131879

[B100] Sakurai K. Zhao S. Takatoh J. Rodriguez E. Lu J. Leavitt A. D. (2016). Capturing and manipulating activated neuronal ensembles with CANE delineates a hypothalamic social-fear circuit. *Neuron* 92 739–753. 10.1016/j.neuron.2016.10.015 27974160 PMC5172402

[B101] Sancataldo G. Silvestri L. Mascaro A. L. A. Sacconi L. Pavone A. F. S. (2019). Advanced fluorescence microscopy for in vivo imaging of neuronal activity. *Optica* 6:758 10.1364/OPTICA.6.000758

[B102] Sbalzarini I. F. (2016). Seeing is believing: quantifying is convincing: computational image analysis in biology. *Adv. Anat. Embryol. Cell Biol.* 219 1–39. 10.1007/978-3-319-28549-8_1 27207361

[B103] Scholz J. LaLiberté C. van Eede M. Lerch J. P. Henkelman M. (2016). Variability of brain anatomy for three common mouse strains. *NeuroImage* 142 656–662. 10.1016/j.neuroimage.2016.03.069 27046115

[B104] Seiriki K. Kasai A. Hashimoto T. Schulze W. Niu M. Yamaguchi S. (2017). High-speed and scalable whole-brain imaging in rodents and primates. *Neuron* 94 1085–1100.e6. 10.1016/j.neuron.2017.05.017 28641108

[B105] Sheng M. Greenberg M. E. (1990). The regulation and function of c-fos and other immediate early genes in the nervous system. *Neuron* 4 477–485. 10.1016/0896-6273(90)90106-P 1969743

[B106] Shimshek D. R. Kim J. Hübner M. R. Spergel D. J. Buchholz F. Casanova E. (2002). Codon-improved Cre recombinase (iCre) expression in the mouse. *Genesis* 32 19–26. 10.1002/gene.10023 11835670

[B107] Shockett P. E. Schatz D. G. (1996). Diverse strategies for tetracycline-regulated inducible gene expression. *Proc. Natl. Acad. Sci. U.S.A.* 93 5173–5176. 10.1073/pnas.93.11.5173 8643548 PMC39217

[B108] Silvestri L. Costantini I. Sacconi L. Pavone F. S. (2016). Clearing of fixed tissue: a review from a microscopist’s perspective. *J. Biomed. Opt.* 21:081205 10.1117/1.JBO.21.8.081205 27020691

[B109] Silvestri L. Muellenbroich M. C. Costantini I. Di Giovanna A. P. Sacconi L. Pavone F. S. (2017). RAPID: real-time image-based autofocus for all wide-field optical microscopy systems. *bioRxiv* [Preprint]. 10.1101/170555

[B110] Silvestri L. Paciscopi M. Soda P. Biamonte F. Iannello G. Frasconi P. (2015). Quantitative neuroanatomy of all Purkinje cells with light sheet microscopy and high-throughput image analysis. *Front. Neuroanat.* 9:68. 10.3389/fnana.2015.00068 26074783 PMC4445386

[B111] Smeyne R. J. Schilling K. Robertson L. Luk D. Oberdick J. Curran T. (1992). Fos-IacZ transgenic mice: mapping sites of gene induction in the central nervous system. *Neuron* 8 13–23. 10.1016/0896-6273(92)90105-M 1730004

[B112] Sørensen A. T. Cooper Y. A. Baratta M. v Weng F. J. Zhang Y. Ramamoorthi K. (2016). A robust activity marking system for exploring active neuronal ensembles. *eLife* 5:e13918. 10.7554/eLife.13918 27661450 PMC5035142

[B113] Spalteholz W. (1914). *Über das Durchsichtigmachen von Menschlichen und Tierischen Präparaten und Seine Theoretischen Bedingungen, Nebst Anhang: Über Knochenfärbung.* North Andover, MA: S. Hirzel.

[B114] Staudt T. Lang M. C. Medda R. Engelhardt J. Hell S. W. (2007). 2,2’-thiodiethanol: a new water soluble mounting medium for high resolution optical microscopy. *Microsc. Res. Tech.* 70 1–9. 10.1002/jemt.20396 17131355

[B115] Susaki E. A. Shimizu C. Kuno A. Tainaka K. Li X. Nishi K. (2020). Versatile whole-organ/body staining and imaging based on electrolyte-gel properties of biological tissues. *Nat. Commun.* 11:1982. 10.1038/s41467-020-15906-5 32341345 PMC7184626

[B116] Susaki E. A. Tainaka K. Perrin D. Kishino F. Tawara T. Watanabe T. M. (2014). Whole-brain imaging with single-cell resolution using chemical cocktails and computational analysis. *Cell* 157 726–739. 10.1016/j.cell.2014.03.042 24746791

[B117] Tainaka K. Kubota S. I. Suyama T. Q. Susaki E. A. Perrin D. Ukai-Tadenuma M. (2014). Whole-body imaging with single-cell resolution by tissue decolorization. *Cell* 159 911–924. 10.1016/j.cell.2014.10.034 25417165

[B118] Tainaka K. Kuno A. Kubota S. I. Murakami T. Ueda H. R. (2016). Chemical principles in tissue clearing and staining protocols for whole-body cell profiling. *Annu. Rev. Cell Dev. Biol.* 32 713–741. 10.1146/annurev-cellbio-111315-125001 27298088

[B119] Tatsuki F. Sunagawa G. A. A. Shi S. Susaki E. A. A. Yukinaga H. Perrin D. (2016). Involvement of Ca2+-dependent hyperpolarization in sleep duration in mammals. *Neuron* 90 70–85. 10.1016/j.neuron.2016.02.032 26996081

[B120] Terleph T. A. Tremere L. A. (2006). “The use of immediate early genes as mapping tools for neuronal activation: concepts and methods,” in *Immediate Early Genes in Sensory Processing, Cognitive Performance and Neurological Disorders*, eds Pinaud R. Tremere L. A. (Boston, MA: Springer). 10.1007/978-0-387-33604-6_1

[B121] Todorov M. I. Paetzold J. C. Schoppe O. Tetteh G. Shit S. Efremov V. (2020). Machine learning analysis of whole mouse brain vasculature. *Nat. Methods* 17 442–449. 10.1038/s41592-020-0792-1 32161395 PMC7591801

[B122] Tomer R. Ye L. Hsueh B. Deisseroth K. (2014). Advanced CLARITY for rapid and high-resolution imaging of intact tissues. *Nat. Protoc.* 9 1682–1697. 10.1038/nprot.2014.123 24945384 PMC4096681

[B123] Tschida K. Michael V. Takatoh J. Han B. X. Zhao S. Sakurai K. (2019). A specialized neural circuit gates social vocalizations in the mouse. *Neuron* 103 459–472.e4. 10.1016/j.neuron.2019.05.025 31204083 PMC6687542

[B124] Tuchin V. v (1997). Light propagation in tissues with controlled optical properties. *J. Biomed. Opt.* 2:401 10.1117/12.281502 23014964

[B125] Ueda H. R. Dodt H. U. Osten P. Economo M. N. Chandrashekar J. Keller P. J. (2020). Whole-brain profiling of cells and circuits in mammals by tissue clearing and light-sheet microscopy. *Neuron* 106 369–387. 10.1016/j.neuron.2020.03.004 32380050 PMC7213014

[B126] Vanni M. P. Chan A. W. Balbi M. Silasi G. Murphy T. H. (2017). Mesoscale mapping of mouse cortex reveals frequency-dependent cycling between distinct macroscale functional modules. *J. Neurosci.* 37 7513–7533. 10.1523/JNEUROSCI.3560-16.2017 28674167 PMC6596702

[B127] Vetere G. Kenney J. W. Tran L. M. Xia F. Steadman P. E. Parkinson J. (2017). Chemogenetic interrogation of a brain-wide fear memory network in mice. *Neuron* 94 363–374.e4. 10.1016/j.neuron.2017.03.037 28426969

[B128] Voigt F. F. Kirschenbaum D. Platonova E. Pagès S. Campbell R. A. A. Kastli R. (2019). The mesoSPIM initiative: open-source light-sheet microscopes for imaging cleared tissue. *Nat. Methods* 16 1105–1108. 10.1038/s41592-019-0554-0 31527839 PMC6824906

[B129] Vousden D. A. Epp J. Okuno H. Nieman B. J. van Eede M. Dazai J. (2015). Whole-brain mapping of behaviourally induced neural activation in mice. *Brain Struct. Funct.* 220 2043–2057. 10.1007/s00429-014-0774-0 24760545

[B130] Wang K. H. Majewska A. Schummers J. Farley B. Hu C. Sur M. (2006). In vivo two-photon imaging reveals a role of Arc in enhancing orientation specificity in visual cortex. *Cell* 126 389–402. 10.1016/j.cell.2006.06.038 16873068

[B131] Wang W. Wildes C. P. Pattarabanjird T. Sanchez M. I. Glober G. F. Matthews G. A. (2017). A light- A nd calcium-gated transcription factor for imaging and manipulating activated neurons. *Nat. Biotechnol.* 35 864–871. 10.1038/nbt.3909 28650461 PMC5595644

[B132] Wilson T. (2010). Spinning-disk microscopy systems. *Cold Spring Harb. Protoc.* 5:db.to88. 10.1101/pdb.top88 21041403

[B133] Winnubst J. Bas E. Ferreira T. A. Wu Z. Economo M. N. Edson P. (2019). Reconstruction of 1,000 projection neurons reveals new cell types and organization of long-range connectivity in the mouse brain. *Cell* 179 268–281.e13. 10.1016/j.cell.2019.07.042 31495573 PMC6754285

[B134] Xie H. Liu Y. Zhu Y. Ding X. Yang Y. Guan J. S. (2014). In vivo imaging of immediate early gene expression reveals layer-specific memory traces in the mammalian brain. *Proc. Natl. Acad. Sci. U.S.A.* 111 2788–2793. 10.1073/pnas.1316808111 24550309 PMC3932903

[B135] Xiu J. Zhang Q. Zhou T. Zhou T. T. Chen Y. Hu H. (2014). Visualizing an emotional valence map in the limbic forebrain by TAI-FISH. *Nat. Neurosci.* 17 1552–1559. 10.1038/nn.3813 25242305

[B136] Yang B. Treweek J. B. Kulkarni R. P. Deverman B. E. Chen C. K. Lubeck E. (2014). Single-cell phenotyping within transparent intact tissue through whole-body clearing. *Cell* 158 945–958. 10.1016/j.cell.2014.07.017 25088144 PMC4153367

[B137] Yang W. Yuste R. (2017). In vivo imaging of neural activity. *Nat. Methods* 14 349–359. 10.1038/nmeth.4230 28362436 PMC5903578

[B138] Ye L. Allen W. E. Thompson K. R. Tian Q. Hsueh B. Ramakrishnan C. (2016). Wiring and molecular features of prefrontal ensembles representing distinct experiences. *Cell* 165 1776–1788. 10.1016/j.cell.2016.05.010 27238022 PMC5708551

[B139] Yu T. Zhu J. Li Y. Ma Y. Wang J. Cheng X. (2018). RTF: a rapid and versatile tissue optical clearing method. *Sci. Rep.* 8 1–9. 10.1038/s41598-018-20306-3 29386656 PMC5792593

[B140] Zipfel W. R. Williams R. M. Webb W. W. (2003). Nonlinear magic: multiphoton microscopy in the biosciences. *Nat. Biotechnol.* 21 1369–1377. 10.1038/nbt899 14595365

